# Analysing WAHIS Animal Health Immediate Notifications to Understand Global Reporting Trends and Measure Early Warning Capacities (2005–2021)

**DOI:** 10.1155/2023/6666672

**Published:** 2023-05-09

**Authors:** Shu-Yu Lin, Daniel Beltran-Alcrudo, Lina Awada, Christopher Hamilton-West, Andrea Lavarello Schettini, Paula Cáceres, Paolo Tizzani, Alberto Allepuz, Jordi Casal

**Affiliations:** ^1^Department de Sanitat I Anatomia Animals, Universitat Autònoma de Barcelona, Bellaterra, Barcelona, Spain; ^2^Regional Office for Europe and Central Asia, Food and Agriculture Organization (FAO), Budapest, Hungary; ^3^World Animal Health Information and Analysis Department, World Organisation for Animal Health (WOAH), Paris, France; ^4^Department of Preventive Veterinary Medicine, Faculty of Veterinary Science, Universidad de Chile, Santiago, Chile

## Abstract

The World Animal Health Information System (WAHIS) from the World Organization for Animal Health (WOAH) is an online reporting system, essential for ensuring the transparency and accuracy of global animal health. One of the WOAH's objectives is to disseminate timely notifications to support countries' efforts to prevent and control the spread of animal diseases. This paper describes the 3,263 exceptional events notified through immediate notifications sent to WOAH from 2005 to February 2021 and their distribution in time and space and by disease. To evaluate the timeliness of reporting, we defined and analysed two periods: the confirmation period (CT), which is the time interval between the disease onset date and the confirmation date, and the notification period (NT), defined as the interval between the disease confirmation and the date of reporting to WOAH. The results showed that (1) the number of events increased over the analysis period; (2) the events were mainly reported for domestic animals and the data provided for wildlife were limited; (3) the official source of disease introduction was often unknown when the event was reported; and (4) the global median CT value was 5 days while the global median NT value was 4 days, with a decreasing trend in both cases over the study period. Divergences were found across world regions and country income categories. This analysis provides interesting insights into the early detection capabilities and transparency of countries, globally and over time.

## 1. Introduction

The increased mobility of animals, their products, and people poses a risk for the transmission of pathogens [1]. Early warning systems aim to detect the incursion of a disease at a relatively early point in time in an attempt to reduce the consequences of the outbreak [2]. This early detection of diseases is essential to successfully and timely manage disease spread at both national and international levels.

The World Organization for Animal Health (WOAH, founded as OIE) was established in 1924 as the reference organization to coordinate and lead the animal health in the world [3] after a huge epidemic of rinderpest that affected Europe and the Americas. One of the WOAH's main objectives is the dissemination of official information related to the presence of animal diseases in countries. It has played a crucial role in timely and transparent data sharing on the global animal health situation, allowing countries at risk to take appropriate action to prevent the spread of transboundary animal diseases (TADs) [4]. Members are committed to submitting notification reports to WOAH related to the diseases listed by the organization and emerging diseases [5]. Other international organizations, such as the Food and Agriculture Organization of the United Nations (FAO), through its Global Animal Disease Information System (EMPRES-i), or regional networks such as the European Commission through the Animal Disease Notification System (ADIS), also publish animal health alerts.

Before 2005, WOAH published early warning reports on a dedicated web page. In 2005, it launched its World Animal Health Information System (WAHIS), which had an early warning component [6]. In 2021, a modernized WAHIS platform [7] was launched, containing all the disease information since 2005, with a technologically advanced yet user-friendly interface, plus new visualization, mapping, and data mining functions.

Since 2005, the disease notification system has consisted of two parts as defined in Chapter 1.1. of WOAH Terrestrial Animal Health Code [5]: (1) the early warning system, which includes immediate notifications to notify alert messages of exceptional epidemiological events, as well as follow-up reports; and (2) the monitoring system comprising six-monthly reports describing the situation of each listed disease in each country and annual reports providing contextual information on national animal populations; zoonotic cases in humans; and resources of animal health national authorities in terms of staff, diagnoses, and vaccine production. WOAH's list of diseases is approved each year by WOAH's General Assembly, based on the criteria identified in the Aquatic and Terrestrial Animal Health Codes, including their international spread, impact on animals and humans, and reliable means of diagnosis [5]. The vast majority of reports are timely and transparently submitted by members to WOAH spontaneously. In complement, WOAH conducts an epidemic intelligence activity to track non-official information from a variety of sources, ask WOAH members for confirmation of additional relevant events, and request the corresponding official reporting [8–10]. FAO, WOAH, and the World Health Organization (WHO), through the Global Early Warning System for Animal Diseases including major Zoonoses (GLEWS+), share information and use their organizational systems to detect threats and verify information via their respective networks [11]. Through these established platforms, transparency is promoted to prevent the spread of cross-border animal diseases, strengthen countries' networks, and provide continuous interaction and cooperation, which can contribute to effectively controlling disease outbreaks.

Based on the notification procedure, the reporting requirement of immediate notifications should follow Article 1.1.3. of the WOAH Terrestrial Animal Health Code [5]. The exceptional epidemiological events related to listed diseases that must be notified through the WAHIS system within 24 hours after confirmation are the following: (a) the first occurrence of a listed disease in a country, zone, or compartment; (b) the recurrence of an eradicated listed disease in a country, zone, or compartment following the final report that declared the event ended; (c) the first occurrence of a new strain of a pathogenic agent of a listed disease in a country, zone, or compartment; (d) the recurrence of an eradicated strain of a pathogenic agent of a listed disease in a country, zone, or compartment following the final report that declared the event ended; (e) a sudden and unexpected change in the distribution or increase in incidence or virulence of, or morbidity or mortality caused by, the pathogenic agent of a listed disease present within a country, zone, or compartment; and (f) the occurrence of a listed disease in an unusual host species. Emerging diseases, as defined by WOAH, should also be reported by WOAH members through the early warning system. The information is mainly submitted electronically through WAHIS where data are organized according to the concepts of events, reports, and outbreaks.

To enable smooth reporting through the online system, national focal points for disease notification receive specific training on the use of this interface [12, 13]. After WOAH receives a report, the information is verified, processed, translated, verified, and publicly displayed in the WAHIS public interface. All WOAH members are also informed in real time of immediate notifications published by the Organization, via emails. As official international warnings, these immediate notifications will allow other countries, trade partners, and other relevant stakeholders to put preventive and control measures in place (e.g., trade restrictions, disease surveillance, and others to protect human and animal health).

An analysis of the alerts for terrestrial animals submitted by countries and published by WOAH over the past few years can provide information on their patterns and help to assess the early warning capacity at both national and global levels. The aim of this study was therefore to analyse the immediate notification reported to WOAH for terrestrial animal diseases between January 2005 and February 2021 to describe the following during the whole period and over time: the diseases reported, the reporting countries and regions, the most common sources, and two early warning indicators—the period between the start of the event and confirmation and the period between confirmation and reporting.

## 2. Materials and Methods

### 2.1. Data Management

Primary data were obtained through WAHIS, extracting data from reports submitted from 1 January 2005 to 8 February 2021. In this period, 3,263 immediate notifications were submitted for 85 listed diseases and 17 emerging diseases of terrestrial animals.

According to the Terrestrial Code [5], countries and territories are required to send an immediate notification report, followed by weekly follow-up reports for a single outbreak or a group of epidemiologically related outbreaks of the diseases that are in the subject of a notification. Reports of an event include information on outbreaks defined as “the occurrence of a disease in an epidemiological unit,” i.e., in an apiary, a backyard, a farm, a forest, a livestock market, a natural park, a slaughterhouse, a village, or a zoo.

The information provided by the immediate notification reports included the susceptible species, the number of affected animals, and spatial and temporal information first described, followed by analysis of information on the source of infection. For a given event, multiple sources of infection can be selected by the reporting country/territory from a drop-down list provided in WAHIS. Reporting countries/territories may also indicate that the source is unknown.

Then, based on the information provided by WOAH members in the immediate notifications, it was possible to identify two main periods of time crucial for the early warning of diseases: (1) the confirmation period (CT), defined as the time interval between the start of the disease event and the date when the disease was confirmed (usually by the national reference laboratory); and (2) the notification period (NT), represented by the time interval between the date of confirmation of the disease and the date when the report was submitted to WOAH. The CT reflects the country's capacity to detect and diagnose a disease event. It may serve as an indicator of early detection and early warning at the national level, and its length will strongly impact the ability to timely control a disease outbreak. On the other hand, NT reveals the timeliness of notification of animal disease events at international level. It may be used as an early warning indicator at the global scale. Any delay in the notification process will affect the effectiveness of regional and global prevention and response. For this analysis, nine events were excluded due to poor data quality on event times.

We assigned the economic status of the countries in four income groups (low, lower-middle, upper-middle, and high income) according to the World Bank classification of countries using the gross national income (GNI) per capita from 2020 [14], and the categorisation of countries within world regions was based on UN classification [15].

### 2.2. Statistical Analysis

We used the Kruskal–Wallis one-way analysis of variance to test if there were significant differences in confirmation/notification times (CT and NT), between years, world regions, or income groups. We evaluated the relationship between CT and NT with Pearson's product-moment correlation coefficient performing all statistical analyses on R 3.6.1 version [16]. Furthermore, we produced the map showing reports' global distribution using QGIS 3.10.3-A [17].

## 3. Results

### 3.1. Distribution of Events by Disease, Year, Animal Category, and Reason for Notification

**Figure 1:**
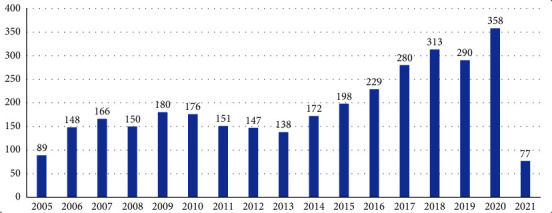
Number of immediate notification reports by submission year (between 1 January 2005 and 8 February 2021).

All the diseases are summarized in supplementary information (Table S.1). The annual number of notification reports increased between 2005 (89 notifications submitted) and 2009 (180 notifications) and then gradually fell to 138 in 2013, before gradually rising to 313 in 2018. This number decreased slightly in 2019 (290 submitted notifications) and reached a new peak in 2020 with 358 notifications. As of 8 February, the number of notifications in 2021 was already 77 (Figure 1).

The most frequently reported diseases are shown in Table 1. Throughout the analysis, infection with high pathogenicity avian influenza virus (poultry) accounted for the most significant number of notifications (560, 17% of the total) with peaks with more than 60 notifications in 2006, 2016, and 2020; African swine fever (ASF) and foot-and-mouth disease (FMD) each represented 11% of the immediate notifications. The annual notification frequency of the diseases shows that each disease had a different trend.

During the period of analysis, the vast majority of events (81%) were reported for domestic animals only, 12% of events were reported for wild animals only, and 7% of events were reported for both domestic and wild animals.

Over the period of analysis, 72% of immediate notifications were submitted for disease recurrence, 15% for disease first occurrence in a zone, 6% for the detection of new strains, 4% for disease first occurrence in a country, 3% for emerging diseases, and less than 1% for a change in disease epidemiology or detection of disease in unusual host.

### 3.2. Geographical Distribution of Events

Most events were notified by countries and territories in Europe (43%), followed by Asia (31%), Africa (15%), the Americas (11%), and Oceania (1%). The three European countries notifying most events were Ukraine (140 events), Germany (126 events), and the Russian Federation (126 events). In Asia, China (People's Rep. of China) notified the highest number of events (160), followed by Israel (105) and Chinese Taipei (Taiwan) (67 events). In Africa and the Americas, only South Africa and the United States of America notified more than 50 events (62 and 67, respectively). Only 37 events were notified by countries and territories of Oceania, 22 of which were from Australia.

**Figure 2:**
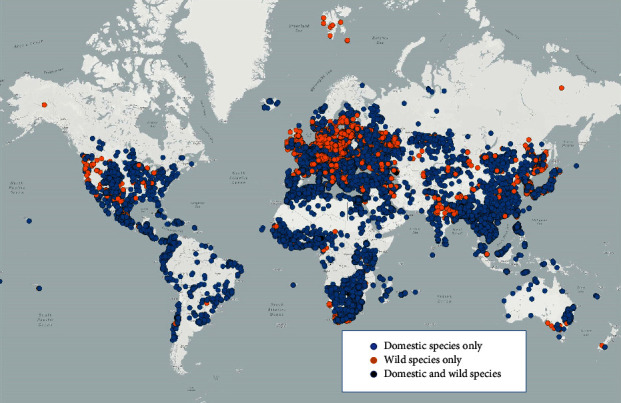
Distribution of outbreaks reported through immediate notifications (between 1 January 2005 and 8 February 2021).

The geographical distribution of the 103,615 outbreaks reported through early warning system during the period of analysis is presented in Figure 2. Out of the outbreaks reported in wildlife, 83% were reported from European countries and territories and 16% were from Asian countries and territories. Other world regions accounted for 1% only.

Notifications came from all but 18 WOAH members (mostly very small countries); in addition, 12 non-WOAH members countries or territories have also reported some immediate notification.

### 3.3. Infectious Sources of Primary Diseases

For 71% of the events considered in this analysis, the reporting country/territory did not select any known source of infection. For the remaining events, the most frequent source selected was “contact with wildlife” (9%); “illegal movement of animals,” “introduction of new live animals,” and “vectors” (7% each); “contact with infected animals at grazing/watering points” (4%); “fomites (humans, vehicles, feed, etc.)” and “legal movement of animals” (3% each); and “airborne spread,” “animals in transit,” and “swill feeding” (1% each). The sources reported for the most frequently reported diseases are shown in Table 2.

### 3.4. Comparison of Differences in Notification Time

Table 3 shows the confirmation period (CT, days between onset of the disease event and laboratory confirmation) and the notification period (NT, days between disease confirmation and reporting to WOAH) along the study period. The median distribution of CT was 5 days, while NT was 4 days. However, there were a high number of events with longer CT and NT periods (the 95th percentiles were 52 and 56 days, respectively). The sum of both periods had median of 11 days. Values of CT reduced over the study period (*p* < 0.0001); in the first 5 years, the median time of CT was 7-8 days, dropping to 3–5 days in the last 5 years. The value of NT was reduced from 4 to 3 days (*p*=0.014). Regarding the most frequently reported diseases, CT and NT periods for ASF became 2 days shorter (for both periods) and, for influenza, 4 and 3 days shorter, respectively. The six most reported diseases have shorter periods than the rest of the diseases (*p* < 0.0001). Moreover, CT and NT showed a positive correlation through a linear regression model, except for FMD, Newcastle disease, and anthrax (Pearson's correlation for all diseases: 0.14, *p* value: <0.0001; values for the most frequent diseases are given in Table S.2).

In terms of world regions (Table 4), European countries showed the shortest CT and NT. For CT, the value above the median was smaller in Europe and Asia than in the other regions. The differences between regions were significant (*p* < 0.0001) except for CT between Africa and the Americas and NT between Europe and Oceania. Regarding the time for laboratory confirmation of the disease (CT), 50% of the European reports were completed within 3 days. In the Americas and Oceania, more than 10% of reports took more than 2 months to get the laboratory confirmation (Table S.3.1).  Table S.3.2 shows that 31% of all reports have an NT of one day. Furthermore, within a week, the notification rate in Europe was as high as 77%, while Africa and the Americas presented the lowest values (52% and 61%, respectively).

On the other hand, there is a clear relationship between the economic level of these countries and both their CT and NT (*p* < 0.0001, both) (Table 5). High-income countries presented shorter periods, showing faster diagnostic procedures and notification time.

## 4. Discussion

One of the WOAH's missions is to improve the early detection and dissemination of information on the occurrence of animal diseases for the global community. It is important to provide countries with timely warnings of new outbreaks of diseases in the world. They allow for improvement of awareness, surveillance, and prevention measures to reduce the global spread of diseases and their consequences for the world economy, livelihoods, animal health, and public health.

Over the years, the number of notifications submitted to WOAH has increased considerably [4], as confirmed also with the results of this study. This increase may be related to a higher awareness of the need to report (i.e., whether through pressure by the international community or by WOAH's efforts to follow up and confirm rumours through the epidemic intelligence activities) that leads to increased transparency. Besides, WOAH has also devoted efforts in training veterinary services in surveillance and notification and establishing a strong network with focal points for disease notification. It may also be partly attributed to improved capacities of veterinary services and laboratories to detect and diagnose, better preparedness of veterinary services, and improved online reporting systems at the national level. This increase in the number of reports could also partly be attributed to the global situation of animal disease epidemics. For example, highly pathogenic avian influenza (HPAI) spread from Asia to Europe between 2004 and 2006, when at least 74 immediate cases were reported. In 2014, a new epidemic wave started with two peaks recorded in 2016 and 2020 with 75 and 140 immediate notifications, respectively. Furthermore, the ASF spread across Asia affecting domestic pigs and wild boar in 2018 [18] that led to 82 immediate reports sent to WAHIS only on that year. Due to these major pandemics, HPAI and ASF were the most frequently notified animal diseases during the study period, as previously described by Mur et al. [6].

The same factors may explain the discrepancies between the number of events reported in different regions of the world. Europe submitted the highest number of reports followed by countries from Asia. Oceania, probably due to the low number of countries and their insularity, had the least number of notifications.

This analysis revealed that most events (81%) were reported only for domestic animals and that almost all outbreaks in wildlife were reported by countries in Europe and Asia. This clearly shows the disparities in surveillance and reporting between animal health sectors as veterinary services are primarily interested in sharing information for the international movement of livestock and their products. Information on livestock and wildlife is collected and managed by different ministries, and in many countries, limited resources are allocated to wildlife surveillance.

Nevertheless, these results have to be interpreted only as global trends. At country and even regional level, the number of reports should not be considered as a measure of the overall animal health status, but rather the result of the country's detection capacity and transparency. For the same reason, a low number (or absence) of immediate notifications for specific countries does not necessarily imply absence of disease. For example, many countries declare the diseases stable in their territories, and the WOAH standards enable them to continue to report these diseases through six-monthly reports [4]. On the other hand, factors influencing the detection capacity might include the surveillance systems in place, the size of the susceptible populations, the ratio between veterinarians and the livestock population, the prevalent production systems (e.g., intensive *vs.* extensive), the clinical expression of the disease, the awareness level of the different stakeholders and their trust in the authorities (which may also vary by disease), whether there is a compensation policy in place, or the laboratory capacity and proficiency. In addition, transparency may vary between diseases, i.e., countries may choose not to report diseases with higher trade consequences or those not notifiable at national level.

The median overall CT was 5 days and tended to decrease over time. This decrease shows an improvement in countries' early detection capabilities. The median global NT was 4 days and similarly tended to decrease over time. This decrease shows an improvement in countries' transparency efforts. However, WOAH Standards (*Aquatic and Terrestrial Animal Health Codes*) require members to submit an immediate notification of an exceptional event within 24 hours of confirming the event; the results of this analysis show that this standard of excellence has not yet been achieved. Timely notification of disease outbreaks through WAHIS enables countries and stakeholders to take action to prevent further disease spread. It also includes the facilitation of safe trade in animals and their products. In this study, NT was within one day for only 31% of reports (38% in Oceania, 36% in Europe, and 32% in Asia).

High-income countries had relatively short CT and NT times. In comparison, low-income countries had a delay in the notification of disease outbreaks. This result could be related to a lack of good management, resourcing, and technical support, which influence disease control strategies [19], and transparency is also likely correlated with the countries' level of income. The low correlation observed between CT and NT indicates that, despite being related, the variables that affect both periods are substantially different.

It is uncertain why some individual events presented a long CT or/and NT period, even more than 60 days. It could be attributed to human filing errors or unawareness or a lack of attention to diseases not considered exceptional. It could also be related to data collection challenges in the field if they are far from the medical/inspection centre [20]. The difficulty to achieve a diagnostic in thedue time can be explained in some case by the shortage of resourcesdevoted by the countries to animal health. In Oceania, the very long CTs in several cases are related to diseases reported after accidental detection of new serotypes or diseases in the frame of some research studies (some of them analysed several months after samples were taken) and the lower number of notifications from the region. CTs can also be very long for new emerging diseases, as surveillance protocols and diagnostic tools are often lacking.

Knowing the potential source of infection and exposure route can help prevent the spread of TADs [21]. This analysis showed that reporting countries were only able to provide source information for 29% of events. The source of an event is frequently identified during trace-back activities. These activities are often not performed in the early stages of event detection but later. This may partially explain why little information is reported to WOAH through immediate notifications and follow-up reports, which are primarily aimed at early warning. In addition, obtaining this information depends on country capacity, and reporting is influenced by heterogeneities in transparency among reporting members. Because of the globalization of the market, international trade has presented a huge increase during the years of study (live pigs, poultry, and meat have increased by more than 80% and live cattle by 40%) [22]. Therefore, the introduction of legal or illegal animals becomes a strong potential source of infection for each disease, and it is likely that the information officially provided by WOAH members on the role of animal movements in the spread of disease through WAHIS early warning system is underestimated. For instance, the source of FMD in Africa was reported as the illegal movement of animals [23]. Wild animals accounted for a large proportion of the source of influenza and rabies, since they act as an important reservoir of the virus.

Only diseases from terrestrial animals have been analysed, and similar studies concerning diseases of aquatic animals would be useful to better understand the importance and efficiency of the immediate notification system. Another limitation of the study is that it does not analyse the extension, the duration, and importance of the different events, nor does it look into specific diseases.

In conclusion, there is a change in the diseases most reported over the period, with an increase in the cases of HPAI and ASF in the last four years. Data collected in this study showed a clear increase over time in terms of the number of disease notifications and a concomitant decrease in the submitting time (both CT and NT). Most of the notified reports were from Europe and Asia, and in general, shorter notification times have been observed in countries with higher incomes. There is still room for improvement by further reducing these periods, applying more resources to the veterinary services, especially in low-income countries. Moreover, these results show an improvement of the transparency indicators at the global level, and they could be useful to review the reporting periods included in Chapter 1.1. of WOAH Terrestrial Animal Health Code.

## Figures and Tables

**Table 1:** Number of immediate notification reports of the top-10 diseases between 1 January 2005 and 8 February 2021.

Disease	2005	2006	2007	2008	2009	2010	2011	2012	2013	2014	2015	2016	2017	2018	2019	2020	2021^‡^	Total
High pathogenicity avian influenza (poultry)	11	60	33	27	12	20	18	15	12	30	52	67	50	42	19	**68**	24	560
African swine fever	1	1	9	3	2	4	1	3	3	12	12	16	43	83	**90**	61	5	349
Foot-and-mouth disease	11	11	23	16	33	24	26	18	19	21	23	14	30	**40**	20	12	2	343
Newcastle disease	**26**	16	12	10	17	16	12	9	9	6	4	7	9	7	15	8	0	183
Low pathogenic avian influenza (poultry) (2006–2021)^†^	0	2	8	10	15	10	10	12	**18**	15	15	15	11	10	11	13	2	177
Influenza A viruses of high pathogenicity (non-poultry including wild birds) (2017-)	0	0	0	0	0	0	0	0	0	0	0	1	52	25	9	**56**	31	174
Bluetongue	1	12	13	14	**20**	8	6	2	2	16	12	17	7	7	5	16	1	159
Anthrax	2	4	2	8	4	5	7	13	6	12	8	**19**	14	17	16	12	0	149
West Nile fever	2	2	0	4	1	9	4	2	1	3	3	2	3	**17**	13	10	0	76
Rabies	0	0	7	7	2	3	5	6	5	2	8	1	7	**9**	5	7	0	74
Others	35	40	59	51	74	77	62	67	63	55	61	70	54	56	87	**95**	12	1018
All	89	148	166	150	180	176	151	147	138	172	198	229	280	313	290	**358**	77	3262

In bold is the year with more reports from each disease. ^†^Dates in parentheses are used to identify situations where a disease was not included in the list of reportable diseases until after 2005 (date before the dash) or was removed from the list of reportable diseases before 2021 (date after the dash). ^‡^As of 8 February.

**Table 2:** Sources of infection for the most reported disease events between 1 January 2005 and 8 February 2021.

Disease	Number of events	Events with known source (%)	Events with unknown source (%)	Airborne spread (%)	Animals in transit (%)	Contact at grazing/watering points (%)	Contact with wildlife (%)	Fomites (humans, vehicles, feed, etc.) (%)	Illegal movement of animals (%)	Introduction of new live animals (%)	Legal movement of animals (%)	Swill feeding (%)	Vectors (%)
High pathogenicity avian influenza (poultry)	560	21	79	1	1	1	**16**	3	3	5	0	0	1
African swine fever	349	22	78	0	0	2	4	6	**11**	5	2	9	2
Foot-and-mouth disease	343	47	53	2	2	16	15	7	**22**	15	7	0	0
Newcastle disease	183	31	69	2	2	1	**15**	6	7	10	2	0	1
Low pathogenic avian influenza (poultry) (2006–2021^†^)	177	13	87	0	0	0	**8**	1	1	2	3	0	0
High pathogenicity influenza (non-poultry including wild birds) (2017-)	174	26	74	1	1	1	**25**	0	1	2	0	0	0
Bluetongue	159	55	45	2	0	4	0	0	2	4	2	0	**52**
Anthrax	149	18	82	0	0	**8**	1	8	0	2	1	1	0
West Nile fever	76	39	61	0	0	0	4	0	0	0	0	0	**37**
Rabies	74	49	51	0	0	0	**23**	0	15	5	0	0	7
Other	1018	29	71	1	1	4	3	2	6	9	5	1	**10**

Sources of infection most frequently reported are highlighted in bold. Some reports indicated more than one source of infection; therefore, the sum of them can be higher than 100%. ^†^Dates in parentheses are used to identify situations where a disease was not included in the list of reportable diseases until after 2005 (date before the dash) or was removed from the list of reportable diseases before 2021 (date after the dash).

**Table 3:** Annual records of the confirmation period (CT), notification period (NT), and total time (CT + NT) in days.

Year	*N*	CT				NT				CT + NT			
		25%	Median	75%	95%	25%	Median	75%	95%	25%	Median	75%	95%
2005	93	3	7	17	54.7	1	4	10	50.4	8	14	30	95.8
2006	156	2	7	17.8	58.5	1	3.5	8.2	57.5	7	14	25.8	92.5
2007	161	2	6	14	43.8	1	4	14	42	6	14	27	91.6
2008	156	2	8	19	78.2	1	5	17.5	77.5	8	17	39.5	140.5
2009	173	2	8	18	64.6	1	5	20	83.8	9	17	40	128.6
2010	182	2	6	13.5	41.9	1	4	9.8	97	5.5	11	28.5	126
2011	159	2	7	13	43	2	6	14.5	60.1	6	14	25	74
2012	131	2.8	7	19	85.5	1	4	17.5	52.5	8	14	35.3	110
2013	143	2	6	14	93	1.5	4	11	79.9	5	11	33.3	184.7
2014	170	2	5	11	59.8	1	4	12.8	48.7	5	11.5	29.3	112.7
2015	206	2	6	18	68.8	1	4	10	41.5	5	13	30	101.5
2016	235	2	6	14	51.5	1	3	8	51.5	5	11	27	93.4
2017	271	2	4	9	39	1	3	9	63	5	10	24	96.4
2018	317	1	4	12	40.2	1	3	7	42.8	4.8	9	20.3	75.5
2019	280	2	5	11	48.9	1	3	10	37.1	5	11	26	87
2020	360	2	4	9	38.3	1	3	9	51.1	4	9	21	73.8
2021	60	1	3	6	14	1	3	6	14.2	3	7	11	26.1
Total	3.25	2	5	13	52	1	4	11	56	2	11	27	99.15

Results include the number of reports, the quartiles, and 95th percentile (the 5th percentile is always zero). ^†^As of 8 February.

**Table 4:** Five regions' records of CT and NT in days.

World region	*N*	CT				NT			
		25%	Median	75%	95%	25%	Median	75%	95%
Africa	473	4	12	27	105	2	7	22	87
Americas	345	4	12	29	92	2	5	18	76
Asia	1,006	2	6	12	45	1	4	11	60
Europe	1,392	1	4	8	31	1	3	7	34
Oceania	37	3	18	54	259	1	3	13	61

Results include the number of reports, the quartiles, and 95th percentile (the 5th percentile is always zero).

**Table 5:** CT and NT records in different economies.

Income groups	*N*	CT				NT			
		25%	Median	75%	95%	25%	Median	75%	95%
High income	1,498	1	4	11	48	1	3	7	45
Upper-middle income	915	2	6	16	60	1	4	13	55
Lower-middle income	722	2	5	14	61	2	5	14	71
Low income	118	5	13	28	109	2	8	23	79

Results include the number of reports, the quartiles, and 95th percentile (the 5th percentile is always zero).

## Data Availability

The data used to support the findings of this study are available from the corresponding author upon request.

## Supplementary Materials

Supplementary Materials Table S.1: list of events notified through WAHIS between 1 January 2005 and 8 February 2021. Table S.2: duration of confirmation period (CT) and notification period (NT) and value of the correlation between them for the most reported diseases. Table S.3.1: confirmation time (CT) duration on five regions. Table S.3.2: notification time (NT) duration on five regions.

## References

[1] Reintjes R., Baumeister H. G., Coulombier D. (2001). Infectious disease surveillance in North Rhine-Westphalia: first steps in the development of an early warning system. *International Journal of Hygiene and Environmental Health*.

[2] Carpenter T. E., Chrièl M., Greiner M. (2007). An analysis of an early-warning system to reduce abortions in dairy cattle in Denmark incorporating both financial and epidemiologic aspects. *Preventive Veterinary Medicine*.

[3] Lightner D. V. (2012). Global transboundry disease politics: the OIE perspective. *Journal of Invertebrate Pathology*.

[4] Cáceres P., Tizzani P., Ntsama F., Mora R. (2020). The world organisation for animal health: notification of animal diseases. *Revue Scientifique et Technique de l’OIE*.

[5] Woah [World Organisation for Animal Health] (2022). Terrestrial animal health Code. https://www.woah.org/en/what-we-do/standards/codes-and-manuals/terrestrial-code-online-access/?id=169&L=1&htmfile=chapitre_notification.htm.

[6] Mur L., Tizzani P., Awada L. (2019). WAHIS, the unique source of official worldwide animal health information, is becoming OIE-WAHIS, a new digital platform. *Frontiers in Veterinary Science*.

[7] Wahis (2023). World animal health information system. https://wahis.woah.org/#/home.

[8] Ben Jebara K., Shimshony A. (2006). International monitoring and surveillance of animal diseases using official and unofficial sources. *Veterinaria Italiana*.

[9] Fanelli A., Awada L., Caceres-Soto P. (2022). Sensitivity of an international notification system for wildlife diseases: a case study using the OIE-WAHIS data on tularemia. *Zoonoses and Public Health*.

[10] Who [World Health Organization] (2022). EIOS leadership and governance. https://www.who.int/initiatives/eios/eios-leadership-and-governance.

[11] Pinto J., Ben Jebara K., Chaisemartin D., De La Rocque S., Abela B. (2011). The FAO/OIE/WHO global early warning system. *FAO Animal Production and Health Paper*.

[12] Cáceres P., Awada L., Barboza P., Lopez-Gatell H., Tizzani P. (2017). The World Organisation for Animal Health and the World Health Organization: intergovernmental disease information and reporting systems and their role in early warning. *Revue Scientifique et Technique de l’OIE*.

[13] Jebara K. B., Cáceres P., Berlingieri F., Weber-Vintzel L. (2012). Ten years’ work on the world organisation for animal health (OIE) worldwide animal disease notification system. *Preventive Veterinary Medicine*.

[14] Hamadeh N., van Rompaey C., Metreau E. (2021). New World Bank country classifications by income level: 2021-2022. https://blogs.worldbank.org/opendata/new-world-bank-country-classifications-income-level-2021-2022.

[15] Unsd [United Nations Statistics Division] (2022). Unsd methodology. https://unstats.un.org/unsd/methodology/m49/.

[16] R Core Team R: A Language and Environment for Statistical Computing. *R Foundation for Statistical Computing*.

[17] Qgis Development Team (2022). QGIS Geographic Information System QGIS Association. https://www.qgis.org.

[18] Awada L., Lambergeon N., Morales R. (2021). Current animal health situation worldwide: analysis of events and trends. https://www.woah.org/en/document/current-animal-health-situation-worldwide-analysis-of-events-and-trends/.

[19] Bucher K., Tellechea D., Caya F., Stratton J. (2020). Implementation of OIE international standards: challenges and opportunities for monitoring. *Revue Scientifique et Technique de l’OIE*.

[20] Ryser-Degiorgis M. P. (2013). Wildlife health investigations: needs, challenges and recommendations. *BMC Veterinary Research*.

[21] Travis D. A., Watson R. P., Tauer A. (2011). The spread of pathogens through trade in wildlife. *Revue Scientifique et Technique de l’OIE*.

[22] Fao (2022). Food and agriculture organization of the UN. https://www.fao.org/faostat/en/#home.

[23] Roberts H., Lopez M. (2012). International disease monitoring, january to march 2012. *The Veterinary Record*.

