# Supplementary material for: Analysing WAHIS Animal Health Immediate Notifications to Understand Global Reporting Trends and Measure Early Warning Capacities (2005–2021)
Source: Transbound Emerg Dis. 2023 May 9;2023:6666672. doi: 10.1155/2023/6666672 (PMC12017132; doi:10.1155/2023/6666672)
Supplement: Supplementary Materials — Table S.1: list of events notified through WAHIS between 1 January 2005 and 8 February 2021. Table S.2: duration of confirmation period (CT) and notification period (NT) and value of the correlation between them for the most reported diseases. Table S.3.1: confirmation time (CT) duration on five regions. Table S.3.2: notification time (NT) duration on five regions. [file 6666672.f1.doc]

1. **Appendices**

**Table S.1. List of events notified through WAHIS between 1 January 2005 and 8 February 2021**

| **Disease** | **N** |
| --- | --- |
| High pathogenicity avian influenza viruses (poultry) (Inf. with) | 560 |
| African swine fever virus (Inf. with) | 349 |
| Foot-and-mouth disease (Inf. with) | 343 |
| Newcastle disease virus (Inf. with) | 183 |
| Low pathogenic avian influenza (poultry) (2006-2021) † | 177 |
| Influenza A viruses of high pathogenicity (Inf. with) (non-poultry including wild birds) (2017-) | 174 |
| Bluetongue virus (Inf. with) | 159 |
| Anthrax | 149 |
| West Nile fever | 76 |
| Rabies virus (Inf. with) | 74 |
| Classical swine fever virus (Inf. with) | 72 |
| Lumpy skin disease virus (Inf. with) | 65 |
| Equine infectious anaemia | 54 |
| Peste des petits ruminants virus (Inf. with) | 50 |
| Sheep pox and goat pox | 47 |
| Rabbit haemorrhagic disease | 43 |
| Bovine spongiform encephalopathy | 37 |
| Rift Valley fever virus (Inf. with) | 37 |
| Influenza A virus (Inf. with) | 32 |
| Melissococcus plutonius (Inf. of honey bees with) (European foulbrood) | 27 |
| Scrapie | 27 |
| African horse sickness virus (Inf. with) | 24 |
| Contagious equine metritis | 24 |
| SARS-CoV-2 in animals (Inf. with) | 24 |
| Equine influenza virus (Inf. with) | 23 |
| Paenibacillus larvae (Inf. of honey bees with) (American foulbrood) | 23 |
| Porcine reproductive and respiratory syndrome virus (Inf. with) | 23 |
| Aethina tumida (Inf. with) (Small hive beetle) (2006-) | 20 |
| Aujeszky's disease virus (Inf. with) | 17 |
| Brucella melitensis (Inf. with) | 17 |
| Mycoplasma mycoides subsp. mycoides SC (Inf. with) (Contagious bovine pleuropneumonia) | 17 |
| Vesicular stomatitis (-2014) | 17 |
| Equine piroplasmosis | 14 |
| Brucella suis (Inf. with) | 13 |
| Burkholderia mallei (Inf. with) (Glanders) | 13 |
| Myxomatosis | 12 |
| Venezuelan equine encephalomyelitis | 12 |
| Fowl typhoid | 11 |
| Leishmaniosis | 11 |
| Equine arteritis virus (Inf. with) | 10 |
| Varroa spp. (Inf. of honey bees with) (Varroosis) | 10 |
| Brucella abortus (Inf. with) | 9 |
| Epizootic hemorrhagic disease virus (Inf. with) | 9 |
| Schmallenberg virus (Inf. with) | 9 |
| Equine encephalomyelitis (Eastern) (2006-) | 8 |
| Equine rhinopneumonitis (-2013) | 8 |
| Porcine epidemic diarrhoea virus (Inf. with) | 8 |
| Bovine tuberculosis (-2018) | 7 |
| Caprine arthritis/encephalitis | 7 |
| Middle East respiratory syndrome coronavirus (inf. with) (2022-) | 7 |
| Q fever | 7 |
| Avian infectious laryngotracheitis | 6 |
| Haemorrhagic septicaemia (Pasteurella multocida serotypes 6:b and 6:e) | 6 |
| Infectious bovine rhinotracheitis/infectious pustular vulvovaginitis | 6 |
| Mycobacterium tuberculosis complex (Inf. with) (2019-) | 5 |
| Tularemia | 5 |
| Avian infectious bronchitis | 4 |
| Bovine anaplasmosis | 4 |
| Dourine | 4 |
| Echinococcosis/hydatidosis | 4 |
| Maedi-visna | 4 |
| Bovine babesiosis | 3 |
| Camelpox (2006-) | 3 |
| Chlamydia abortus (Inf. with) (Enzootic abortion of ewes, ovine chlamydiosis) | 3 |
| Enzootic bovine leukosis | 3 |
| Equid herpesvirus-1 (Inf. with) (Equine rhinopneumonitis) (2014-) | 3 |
| New world screwworm (Cochliomyia hominivorax) | 3 |
| Pullorum disease | 3 |
| Transmissible gastroenteritis | 3 |
| Avian chlamydiosis | 2 |
| Contagious agalactia | 2 |
| Contagious caprine pleuropneumonia | 2 |
| Duck virus hepatitis | 2 |
| Heartwater | 2 |
| Infectious bursal disease (Gumboro disease) | 2 |
| Mycoplasma gallisepticum (Avian mycoplasmosis) (Inf. with) | 2 |
| Old world screwworm (Chrysomya bezziana) | 2 |
| Paratuberculosis | 2 |
| Trypanosomosis (tsetse-transmitted) (-2021) | 2 |
| Acarapis woodi (Inf. of honey bees with) | 1 |
| Avian mycoplasmosis (M.synoviae) (2006-) | 1 |
| Botulism (-2014) | 1 |
| Bovine genital campylobacteriosis | 1 |
| Bovine viral diarrhoea (2006-) | 1 |
| Ehrlichia canis (Inf. with) | 1 |
| Equine encephalomyelitis (Eastern and Western) (-2005) | 1 |
| Equine encephalomyelitis (Western) (2006-) | 1 |
| Equine encephalosis virus (Inf. with) | 1 |
| Hendra viruses (Inf. with) | 1 |
| Malignant catarrhal fever (wildebeest only) (2006-2008) | 1 |
| Monkey pox | 1 |
| Mycobacterium tuberculosis (Inf. with) (-2017) | 1 |
| Ovine epididymitis (Brucella ovis) | 1 |
| Pigeon rotavirus | 1 |
| Postweaning Multisystemic Wasting Syndrome (PMWS) | 1 |
| Pox viruses (other than those listed by the OIE) (Inf. with) (2011-) | 1 |
| Streptococcus suis (Inf. with) | 1 |
| Surra (Trypanosoma evansi) | 1 |
| Swine vesicular disease (-2014) | 1 |
| Trichinella spp. (Inf. with) | 1 |

† Dates in parentheses are used to identify situations where a disease was not included in the list of reportable diseases until after 2005 (date before the dash) or was removed from the list of reportable diseases before 2021 (date after the dash)

**Table S.2. Duration of confirmation period (CT), notification period (NT) and value of the correlation between them for the most reported diseases (values in brackets indicate the interquartile range).**

|  |  |  | **Median** | | | **Correlation Test (CT ~ NT)** | |
| --- | --- | --- | --- | --- | --- | --- | --- |
| **Disease** |  | **N** | | **CT** | **NT** | **Coefficient** | **p-value** |
| **Influenza** |  | 887 | | 4 (2-9) | 3 (1-6) | 0.12 | 0.0004 |
| **African swine fever** |  | 339 | | 2 (1-6) | 2 (1-5) | 0.29 | 5.249e-08 |
| **Foot and Mouth disease** |  | 267 | | 6 (3-12) | 3 (1-10) | 0.07 | ns* |
| **Newcastle disease** |  | 173 | | 7 (2-14) | 4 (1-10.5) | 0.08 | ns |
| **Bluetongue** |  | 147 | | 6 (2-21) | 5 (1-14.5) | 0.23 | 0.004 |
| **Anthrax** |  | 133 | | 4 (2-8) | 4 (1-9) | -0.02 | ns |
| **Other diseases** |  | 766 | | 9 (3-22) | 6 (2-20) | -0.005 | ns |
| **All diseases** |  | 2,973 | | 5 (2-13) | 4 (1-11) | 0.14 | 3.729e-14 |

ns: not significant

**Tables S.3. Confirmation time (CT) and Notification time (NT) durations on five world regions**

| Table S.3.1. Confirmation time (CT) duration on five regions. | | | | | | | |
| --- | --- | --- | --- | --- | --- | --- | --- |
| World region | N | 0-1 day | 2-3 days | 4-7 days | 8-30 days | 31-60 days | > 60 days |
| Africa | 473 | 13% | 11% | 18% | 36% | 13% | 9% |
| Americas | 345 | 14% | 8% | 12% | 42% | 14% | 11% |
| Asia | 1,006 | 16% | 20% | 25% | 30% | 5% | 4% |
| Europe | 1,392 | 33% | 17% | 22% | 23% | 4% | 2% |
| Oceania | 37 | 22% | 5% | 11% | 30% | 8% | 24% |
| Total | 3,253 | 23% | 16% | 21% | 29% | 6% | 5% |

| Table S.3.2. Notification time (NT) duration on five regions. | | | | | | | |
| --- | --- | --- | --- | --- | --- | --- | --- |
| World region | N | 0-1 day | 2-3 days | 4-7 days | 8-30 days | 31-60 days | > 60 days |
| Africa | 473 | 18% | 15% | 19% | 29% | 9% | 10% |
| Americas | 345 | 22% | 19% | 20% | 26% | 7% | 7% |
| Asia | 1,006 | 32% | 18% | 18% | 22% | 6% | 5% |
| Europe | 1,392 | 36% | 21% | 20% | 17% | 4% | 2% |
| Oceania | 37 | 38% | 14% | 14% | 22% | 8% | 5% |
| Total | 3,253 | 31% | 19% | 19% | 21% | 6% | 4% |
